# Preparation of Cellulose Films from Sustainable CO_2_/DBU/DMSO System

**DOI:** 10.3390/polym11060994

**Published:** 2019-06-04

**Authors:** Longming Jin, Jianyun Gan, Gang Hu, Long Cai, Zaiquan Li, Lihua Zhang, Qiang Zheng, Haibo Xie

**Affiliations:** Department of Polymer Materials & Engineering, College of Materials & Metallurgy, Guizhou University, West Campus, Huaxi District, Guiyang 550025, China; lmjin5369@163.com (L.J.); gs.jygan18@gzu.edu.cn (J.G.); richardhugang@126.com (G.H.); cailong1015@163.com (L.C.); lzaiquan483@163.com (Z.L.); lhzhang1@gzu.edu.cn (L.Z.)

**Keywords:** CO_2_, cellulose dissolution, cellulose films, regeneration

## Abstract

Cellulose films are regarded as sustainable materials having wide applications in food packaging, separation, etc. Their preparation substantially relies on sufficient dissolution. Herein, various celluloses adequately dissolved in a new solvent system of carbon dioxide,1, 8-diazabicyclo [5.4.0] undec-7-ene and dimethyl sulfoxide (CO_2_/DBU/DMSO) were made in to films using different regeneration reagents. The films regenerated from ethanol and methanol presented homogeneous and smooth surfaces, while those from 5 wt % NaOH (aq.) and 5 wt % H_2_SO_4_ (aq.) showed rough surfaces, as analyzed using scanning electron microscopy (SEM) and atomic force microscopy (AFM). The films regenerated from 5 wt % NaOH (aq.) and 5 wt % H_2_SO_4_ (aq.) rendered cellulose II structures, while those regenerated from alcohols had amorphous structures as evidenced using fourier transform infrared spectroscopy (FT-IR) and X-ray diffraction (XRD) results. The films made of microcrystalline cellulose had a good light transmittance of about 90% at 800 nm with a tensile strength of 55 MPa and an elongation break of 6.5%, while those from wood pulp cellulose demonstrated satisfactory flexibility with a tensile strength of 91 MPa and an elongation break of 9.0%. This research reports a simple, environmental, and sustainable method to prepare cellulose films of good mechanical properties.

## 1. Introduction

The increasing consumption of crude oil highlights the effects of the concerns of human beings being prioritized over resource availability and environmental crisis. Renewable biomass thus becomes a mainstream research topic [1]. Shifting the exploitation of currently depletive crude oil to sustainable bio-based resources will not only bring a new blueprint for resource richness but also benefits our environment. Cellulose, an important natural biopolymer, is the main biomass with distinctive properties including renewability, high thermal stability, and biodegradability [2,3]. Cellulose films have great potential for applications in food packaging, the biomedical field, conductive materials, and chiral resolution [2,4,5,6,7]. The first challenge for the preparation of cellulose films is the successful dissolution of cellulose by solvents. It was identified that the regeneration method had a significant effect on the chemical-physical structure of the cellulosic film and also on the material properties. Therefore, new technologies for forming cellulose films have appeared, along with the development of various dissolution strategies. The various solvents capable of dissolving cellulose include *N*-methylmorpholine *N*-oxide (NMMO) [8], dimethylsulfoxide–tetrabutylammonium fluoride (DMSO–TBAF) [9], *N*,*N*-dimethylacetamide–lithium chloride (DMAc–LiCl) [10], NaOH/Urea aqueous solutions, and ionic liquids (ILs), etc. [11,12]. Qi et al. dissolved cellulose in 7 wt %NaOH/12 wt% urea aqueous solution to prepare regeneration cellulose films with *M_η_* = 11.1×10^4^. The films had good transmittance of 90% at 800 nm, and the tensile strength reached about 100 MPa [13]. Zhang etal. used molten salt hydrate of lithium bromide (LiBr) to prepare regenerated cellulose films from kraft pulp. The maximum elongation break and tensile strength reached 26% and 67 MPa for films regenerated from 65 wt % LiBr [14]. Pang et al. used ionic liquid 1-ethyl-3-methylimidazolium acetate to dissolve different celluloses (pine, cotton, bamboo, MCC) at 5 wt % for film preparations, and the tensile strengths and elongation breaks were 69–120 MP and 6–8%, respectively [15]. Zheng et al. investigated preparation of cellulose films from cellulose (DP~400) dissolved in 1-butyl-3-methylimidazolium chloride ([Bmim][Cl]), 1-allyl-3-methylimidazolium chloride ([Amim][Cl]), and 1-ethyl-3-methylimidazolium acetate ([Emim][Ac]) solvent arrangements. The tensile strength and transmittance of films were in the order of [Amim][Cl] > [Bmim][Cl] > [Emim][Ac], and the maximum tensile strength and transmittance reached 152±30 MPa and 90±3%, respectively [16]. Wang et al. used choline chloride/urea (ChCl/urea), a deep eutectic solvent (DES), as both a solvent and a plasticizer to prepare cellulose film with a high elongation break (34.88%) [17].

Carbon dioxide (CO_2_), one of the main greenhouse gases, has an undesirable impact on the ecological environment; nevertheless, it can be used as a main C1 resource through capture and storage [18,19]. Recently, a sustainable CO_2_-based solvent system for cellulose dissolution has been reported almost simultaneously by Xie et al. [20] and by Zhang et al. [21] Compared with the existing methods, this approach is a novel method in terms of its efficiency and sustainability. The potentials of this approach for developing cellulosic materials have been reported in synthesizing cellulose esters [22,23,24], cellulose grafted polylactic acid (PLA) [25], but regeneration of cellulose films under this system remains unreported.

**Figure 1:**
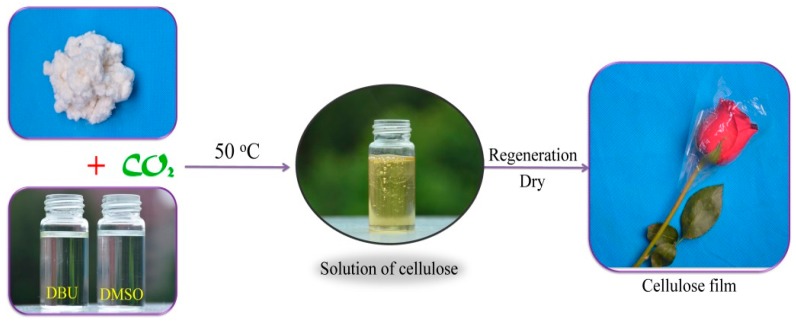
Flowchart of the experiment.

In this study, in order to identify the potential of cellulose film preparation of the newly developed CO_2_/DBU/DMSO solvent system for cellulose, a series of cellulose films were prepared after the successful dissolution of cellulose in CO_2_/DBU/DMSO using different regeneration solvents (Figure 1). The effect of regeneration bath and molecular weight of cellulose on the properties of cellulose films was studied through the surface morphology, the roughness of the regenerated cellulose films was characterized using SEM and AFM, and the crystalline structure of films was characterized using FT-IR and XRD. The light transmittance and tensile measurements were implemented on the films from different regeneration solvents, transmittance, and tensile measurements. The authors suggest that this study presents a simple, sustainable, efficient, and novel dissolving system for preparing cellulose films with new practical applications.

## 2. Materials and Methods

### 2.1. Materials

Microcrystalline cellulose (MCC, DP = 220, particle size: ∼50 μm), cotton cellulose (CPC, DP = 350, particle size: ∼50 μm), wood pulp cellulose (DP = 500, particle size: ∼50 μm), and 1, 8-diazabicyclo [5.4.0] undec-7-ene (DBU) were purchased from Aladdin Reagents Co., Ltd. (Shanghai, China). Methanol, ethanol, and dimethyl sulfoxide (DMSO) of analytical grade were purchased from Kermel Reagents Co., Ltd. (Tianjin, China). DMSO was dried using 4 A molecular sieves prior to the experiments. Carbon dioxide (CO_2_) was supplied by Guiyang Shenjian Gas Co., Ltd. (Guizhou, China) with a purity of >99.99%. Sulfuric acid (H_2_SO_4_, 98%) was purchased from Chuandong Chemical Co., Ltd. (Chongqing, China). Sodium hydroxide (NaOH, analytical grade) was purchased from Sinopharm Chemical Reagent Co., Ltd. (Shanghai, China).

### 2.2. Dissolution of Cellulose and Preparation of Films

**Scheme 1:**
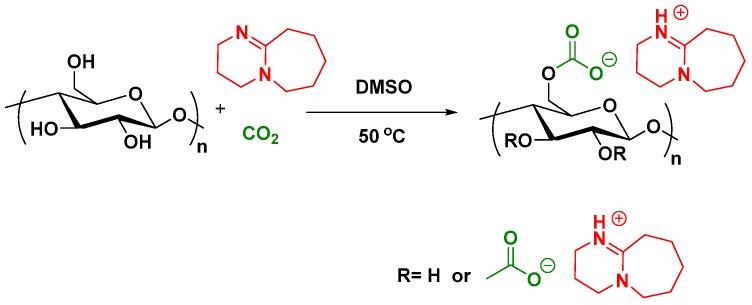
Dissolutionmechanism of cellulose.

Cellulose (5%) was suspended in the mixed solution of 1,8-diazabicyclo[5.4.0]undec-7-ene (DBU, 1 eq. with respect to −OH groups of cellulose) and DMSO in a stainless-steel reactor (Scheme 1). The reactor with 0.5 MPa of CO_2_ pressure was placed in an oil bath at 50 °C after replacing the air with CO_2_ three times. Complete dissolution was achieved within 5 h for all kinds of celluloses except for MCC, which required 3 h for a clear solution. Cellulose solutions were subsequently degassed using a high-speed centrifuge at 10,000 rpm for 5 min and then cast onto clean glass plates and screed using a glass rod with a definite radius copper wire at each end. Transparent cellulose gels formed immediately after the glass plates were immersed in regeneration baths. Twelve-hour immersion was applied to thoroughly replace such impurities as DBU and DMSO in the gels. Finally, the gels were spread flat and turned around on a glass plate at a temperature of 50 °C until the surfaces became elastic. This was followed by being squeezed between two glass plates with 25 kg weight at room temperature for about 24 h. In order to investigate the correlation between the regeneration solvents and performances of cellulose films, the regeneration solvents selected included ethanol, methanol, sodium hydroxide solution (5% wt), and sulphuric acid solution (5% wt). Cellulose films were analyzed on their tensile strengths, crystallinities, morphological features, and transmittances. The optimized regeneration conditions were further applied to CPC and wood pulp cellulose to investigate the impacts of DP and source of cellulose on film properties.

### 2.3. Structure Characterization

The thickness of the regenerated cellulose films was measured using a micrometer at 5 locations on each film, and the mean values were used for subsequent calculations of mechanical properties.

XRD spectra of regenerated cellulose films and raw cellulose specimens were recorded on a X’pert Pro-1 diffractometer equipped with a Cu Kα radiation source (λ = 1.54 Å), operated at 40 kV and 40 mA. Scans were collected from 2 θ = 5–75° with a step size of 0.016° and a scan speed of 0.208°/s [26,27]. The cellulose crystallinity index (CrI) was estimated according to the following empirical Equation (1) [28]:

(1) $$\mathrm{CrI}=\frac{\left[I_{t}\;-I_{a}\right]}{I_{t}},$$

where I_t_ is the total intensity of the (2 0 0) peak for cellulose I 2θat22.7° and of the (0 2 0) peak for cellulose II 2θat 21.7°, and I_a_ is the amorphous intensity 2θat 18° for cellulose I and 2θat 16° forcellulose II.

Fourier transform infrared spectroscopy (FTIR) analyses of the prepared cellulose films were conducted using a Bruker Tensor 27 spectrometer under ATR mode. All the films were dried in vacuum at 40 °C for 12 h. Spectra were recorded over the range of 400–4000 cm^−1^ with a resolution of 4 cm^−1^.

### 2.4. Morphological Observation

The surface morphologies of the regenerated cellulose films were characterized using a JEOL 7800F scanning electron microscopy (SEM) instrument. A sample was fixed with two-side tape on a sample table and then coated with gold for 15 min using a vacuum sputter coater prior to observation. Images were acquired with 10 kV accelerating voltages. Anenergy-dispersive X-ray spectroscopy (EDS) was employed to detect the proportion of elemental distributions under free retrieval element mode.

The surface roughness of the films was determined using atomic force microscopy (AFM) with a Bruker Dimension ICON instrument under tapping mode. AFM images were taken with a scan rate of 1.00 Hz using tapping mode. Scan sizes were 5–10μm with 512 scans on each scanning line.

### 2.5. Strength of Cellulose Films

Tensile strengths of the regenerated cellulose films were measured on a universal testing machine (UTM 4204, Shenzhen SANS test machine, Shenzhen, China). The test mode was by a Chinese standard (GB/T1040.3-2006), whereby the strain rate is 4mm/min, the grip space is 30 mm, the width of each sample is 15 mm, and thickness is 0.015–0.025 mm. The measurements were conducted at 25 °C with a relative humidity of 50 ± 5%. Five replicates for every specimen were tested, and the mean values were reported.

### 2.6. Transparency

Transmittances were determined using an Ultrospec 8000 (GE Healthcare, Chicago, IL, USA) spectrophotometer. Spectra were recorded over the range of 200–800 nm with a resolution of 1 nm. Transmittances were measured at 5 locations of each film, and the average values were reported.

## 3. Results and Discussion

### 3.1. Crystallinity of Cellulose Films

**Figure 2:**
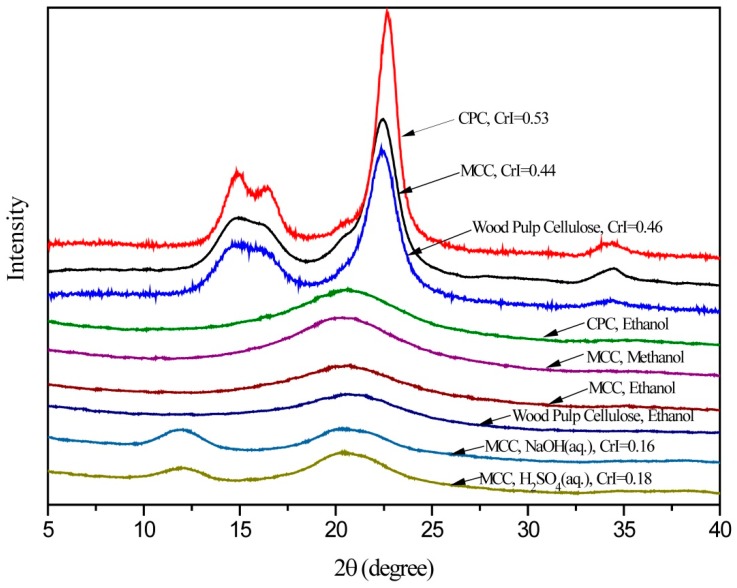
XRD patterns of raw cellulose powders and regenerated cellulose films.

Figure 2 shows XRD patterns of the raw cellulose powders and cellulose films in this research. These patterns shed light on how dissolution and regeneration had affected the crystalline structures of the cellulose. The CrIs of the cellulose and films were calculated using High Score software (PANalytical B.V. Almelo, Netherlands), and the results are displayed in Table 1. Typical diffractions of CPC are observed at 2θ = 14.8°, 16.3°, and 22.6°, which correspond to the (110), and (200) planes, respectively, belonging to the I_β_-rich raw cellulose [29]. For the Iα-rich MCC and wood pulp cellulose, three distinct diffractions, (100), (010), and (110), are observed at 2θ = 14.6°, 16.9°, and 22.7°, respectively [30]. All kinds of raw cellulose have a similar weak peak at 34.7° corresponding to (004) crystal planes of cellulose I [31]. These diffraction peaks disappear on the XRD for cellulose films regenerated from ethanol and methanol, and broad peaks at about 2θ=21° show up instead. Such changes imply that cellulose I completely transformed to amorphous cellulose following the alcohol-based regeneration process, and the XRD patterns of the amorphous cellulose film agree with previous research in the literature [32,33,34]. On the other hand, the cellulose films regenerated from 5 wt % NaOH(aq.) and 5 wt % H_2_SO_4_(aq.) show similar diffractions, besides the weak peaks at around 2θ = 12.1°. The diffractions are features of cellulose II crystal structures [13,15], indicating that the conformation of cellulose molecular chains was affected by the regeneration environment during the regeneration process. The aqueous baths provided settings that induce cellulose re-crystallization during the reformation of the broken inter- and intramolecular hydrogen bonds among cellulose molecules [35].

### 3.2. FT-IR of Cellulose and Regenerated Films

**Figure 3:**
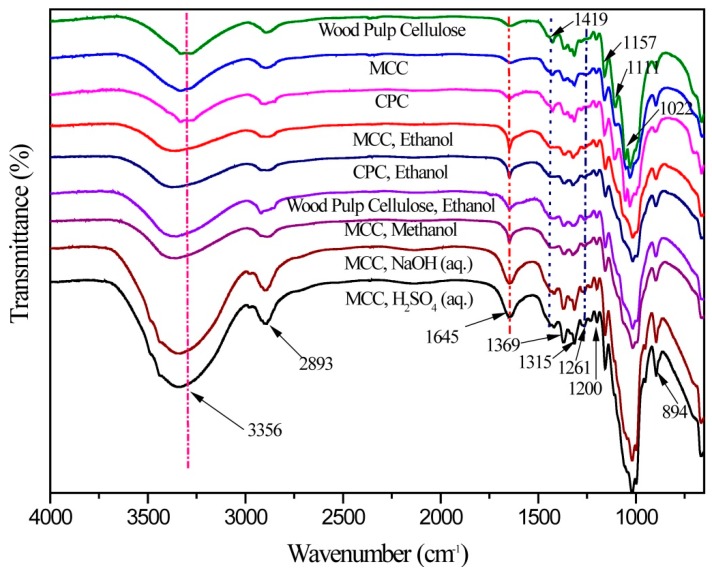
FT-IR of raw cellulose and regenerated cellulose films.

Figure 3 presents the FT-IR spectra of raw cellulose powders and regenerated films in this research. All the spectra exhibit similar characteristic absorptions at 3356, 2893, 1645, 1419, 1369, 1315, 1261, 1200, 1157, and 1022 cm^−1^, which are associated with cellulose. The bands at 3356 cm^−1^ are attributed to the −OH stretching vibration and the bands at 2896 cm^−1^ to CH-stretching. The peaks at 1645cm^−1^ are probably due to small amounts of carbonyl groups introduced by a variety of preparation, processing and purification steps [36]. Absorbance in the fingerprint regions at 894 and 606 cm^−1^ is a feature of the anhydroglucose unit. A shoulder at 1157 cm^−1^ may be attributed to C-O-C pyranose ring skeletal vibrations in cellulose. The strong bands at 1023 cm^−1^ are assigned to the characteristic C-O-C stretching [37]. The absorptions at 1419cm^−1^ are characteristics of the CH_2_ symmetrical bending vibrations, and the absorptions at 894 cm^−1^ are in response to the changes in molecular conformations due to rotation about the β-(1/4)-d-glucosidic linkages. Normally, the absorption bands at 1419, 1157, 1111, and 897 cm^−1^ are used to measure the crystallinity of cellulose. For the raw MCC, CPC, and wood pulp cellulose, sharp absorptions appear at 1419cm^−1^ and weak ones at 897 cm^−1^. On the other hand, broad absorptions show up at 1419cm^−1^ for all cellulose films with increased intensity of absorptions at 897 cm^−1^, suggesting lower crystallinity for the regenerated films. In addition, decreased intensities are observed for the peaks at 1419, 1157, 1111, and 1022 cm^−1^ after the regenerations.

The broad absorptions in the 3000–3600 cm^−1^ region, due to the −OH stretching vibrations, could reflect changes of the hydrogen bonds. Narrow peaks at 3340 cm^−1^ for native cellulose are caused by the regular arrangements of intramolecular and intermolecular hydrogen bonds [32]. After regenerations, the regularities of the hydrogen bonds were disturbed, and the peaks shifted to a higher wave number at 3356 cm^−1^ with bands broadened. The films regenerated from 5 wt % NaOH (aq.) and 5 wt % H_2_SO_4_ (aq.) presented all peaks at the same wave numbers but stronger absorptions, which may be due to the presence of crystalline cellulose II. Therefore, it can be postulated that the cellulose films regenerated from ethanol and methanol baths were mainly composed of amorphous cellulose, but the films from aqueous baths were composed of both cellulose II and amorphous cellulose.

### 3.3. Topography of Films

**Figure 4:**
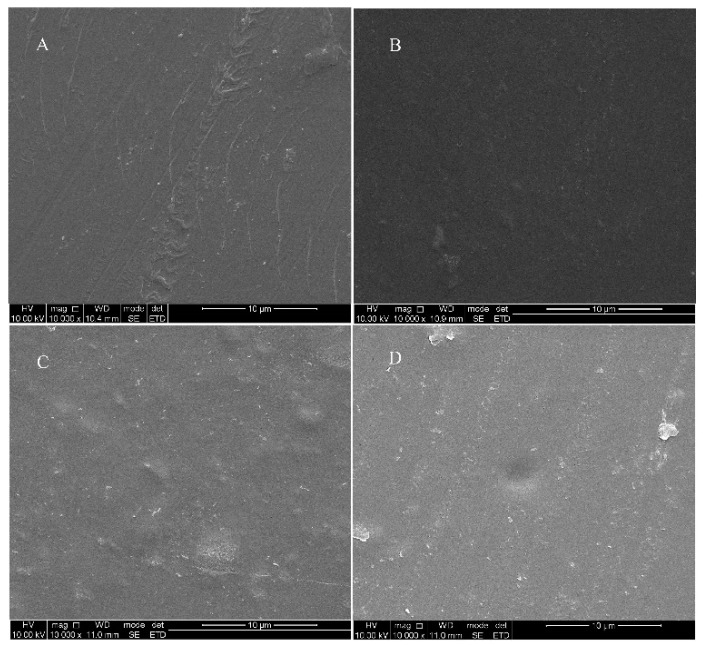
SEM micrographs of regenerated microcrystalline cellulose (MCC)films from different regeneration baths: (**A**) methanol; (**B**) ethanol; (**C**) 5 wt % NaOH (aq.); (**D**) 5 wt % H_2_SO_4_ (aq.).

The morphologies of the obtained cellulose films were characterized by SEM and AFM. Figure 4 and Figure 6 show typical SEM and AFM images of MCC films regenerated from ethanol (A), methanol (B), 5 wt % NaOH (C), and 5 wt % H_2_SO_4_ (D) aqueous solutions. The SEM images at 10,000 magnification implies that the regeneration media had a certain influence on the surface morphology of the regenerated films. The cellulose films prepared in ethanol and methanol baths (Figure 4A,B) displayed homogeneous and smooth surfaces. The film regenerated from an ethanol bath presented a tighter surface mainly due to lower volatilization of ethanol during the dry process as compared with that of methanol. However, Figure 4C,D present rough and ragged SEM images for cellulose films regenerated from aqueous solutions of NaOH (5 wt %) and H_2_SO_4_ (5 wt %), respectively. These differences suggest that the alcohols were better regeneration solvents than aqueous baths for films to have good appearances. All the SEM images present some small nodules and certain contours, which may be derived from strong hydrogen bonds of cellulose [38].

**Figure 5:**
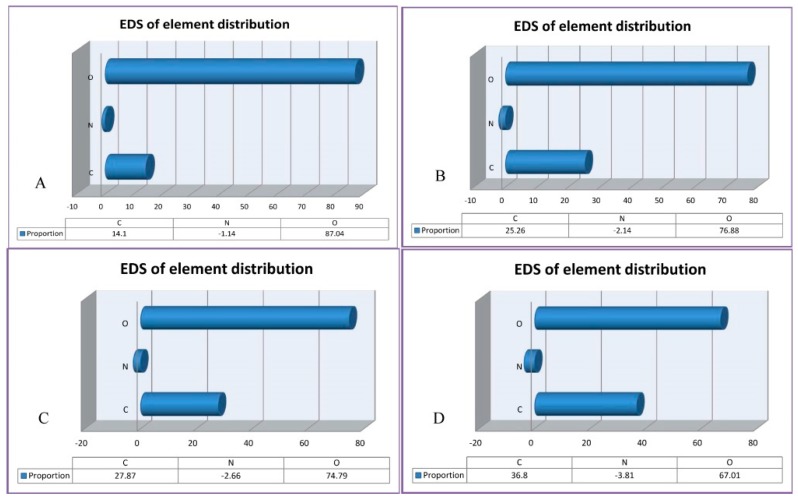
Energy-dispersive X-ray spectroscopy (EDS) results of regenerated MCC films from different regeneration baths: (**A**) methanol; (**B**) ethanol; (**C**) 5 wt % NaOH (aq.); (**D**) 5 wt % H_2_SO_4_ (aq.).

In order to confirm the removal of DBU, the regenerated films were analyzed using energy-dispersive X-ray spectroscopy (EDS), and the results are shown in Figure 5. Only carbon, nitrogen and oxygen were selected to be measured for the proportion of elemental distribution. As presented in Figure 5, nitrogen was negligible for all four films, which means that that DBU was completely removed during the regeneration processes.

**Figure 6:**
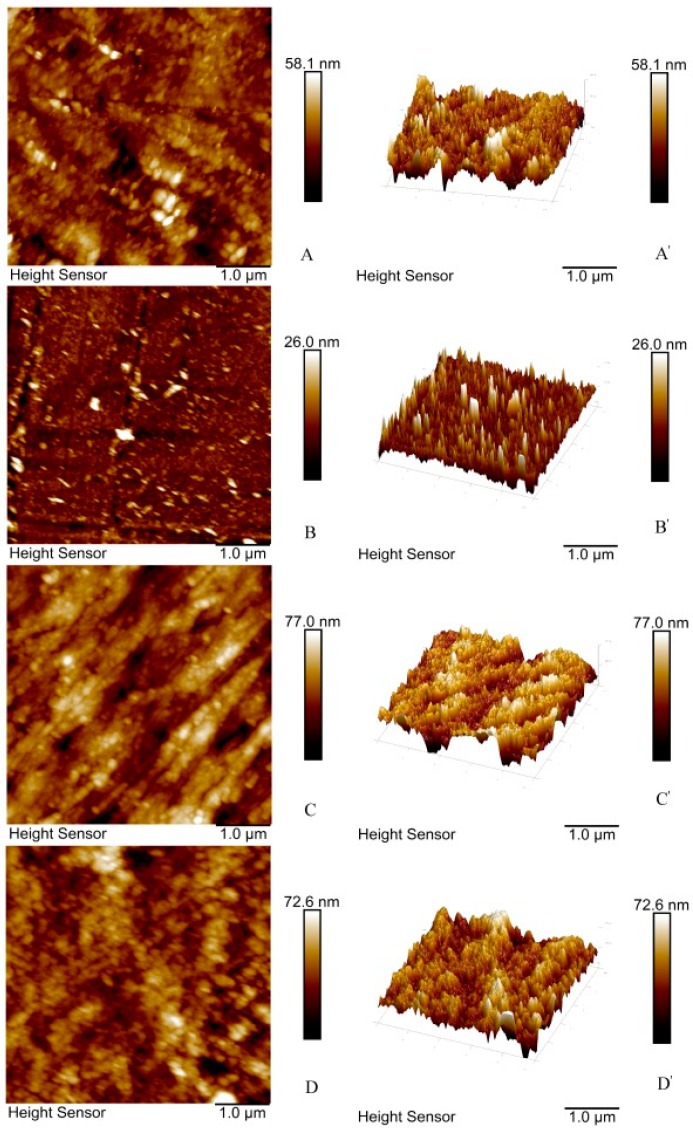
Atomic force microscopy (AFM)micrographs of regenerated MCC films from different regeneration baths: (**A**) methanol; (**B**) ethanol; (**C**) 5 wt % NaOH (aq.); (**D**) 5 wt % H_2_SO_4_ (aq.).

The roughness of the film affects its applications, especially in the field of optics. The higher resolution of AFM images (Figure 6) also shows the determined structural roughness of cellulose films. All the films displayed light, pot-distributed light-pot distributed morphology; the lightness indicates the altitudes of the film surfaces. The mean roughness determined for the four types of regenerated cellulose films are R_a_ (A) =4.03 nm, R_a_ (B) =2.10 nm, R_a_ (C) =7.73 nm, and R_a_ (D)=6.92 nm. It is evident that the roughness of films regenerated in an ethanol bath (Figure 6B) was much lower than the ones regenerated using the other three approaches, which was consistent with the results from the SEM. It can also be observed that the films prepared in aqueous baths exhibited rougher appearances, which may be due to the re-crystallization of cellulose induced by water [38] and the reaction between the cellulose-based carbonate anion [39] and acid/base.

### 3.4. Tensile Strength

**Figure 7:**
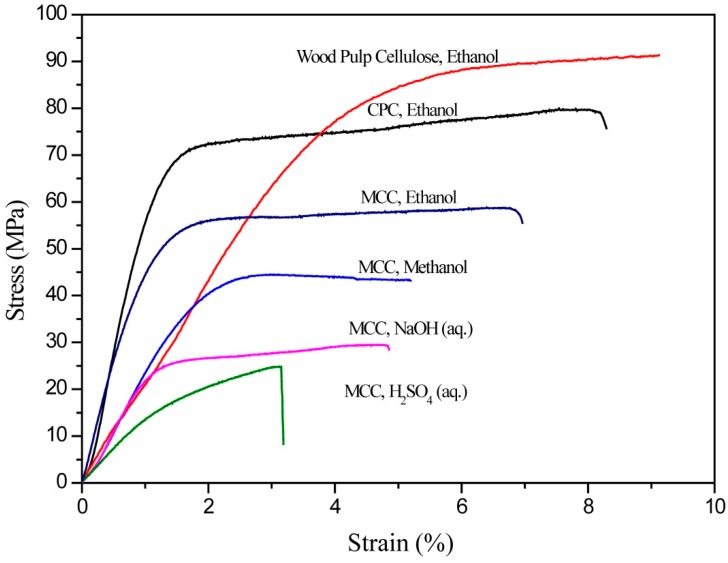
Stress–strain curves of films.

Mechanical properties are important indicators for shaping appropriate applications of polymeric materials. Figure 7 and Table 1 show the stress-strain curves of the CPC and wood pulp cellulose films regenerated from ethanol and MCC films from alcohol-based and aqueous baths. The MCC film regenerated from 5 wt % NaOH (aq.) became rather brittle and fragile with a tensile strength of 25 MPa, while the films from 5wt % H_2_SO_4_ (aq.) tended to be resilient with the lowest tensile strength of 21 MPa. The MCC film prepared from ethanol exhibited a higher tensile strength of 55 MPa and an elongation break of 7%. It can be inferred that the mechanical properties of these MCC films mainly depend on tightness and homogeneity, roughness, and hole defects other than crystalline structures. The MCC, CPC, and wood pulp cellulose films regenerated from ethanol baths displayed increased tensile strengths and elongation breaks. The wood pulp cellulose film exhibited an excellent tensile strength of 91 MPa and an elongation break of 9% due to its greater DP. Compared with the MCC and CPC films, the wood pulp cellulose film showed a good flexibility along with lower Young’s modulus (Table 1). Although the mechanical properties of cellulose films mainly depend on their crystallinity and DP, crystallinity becomes more decisive when the DP reaches a critical point (DP = 450) [15]. The XRD results have demonstrated that all the films from the ethanol baths were composed of amorphous cellulose, their tensile strengths were thence determined almost solely by DP (under 450), so were the tensile strengths of MCC and CPC films. [40].

### 3.5. Transmittance

**Figure 8:**
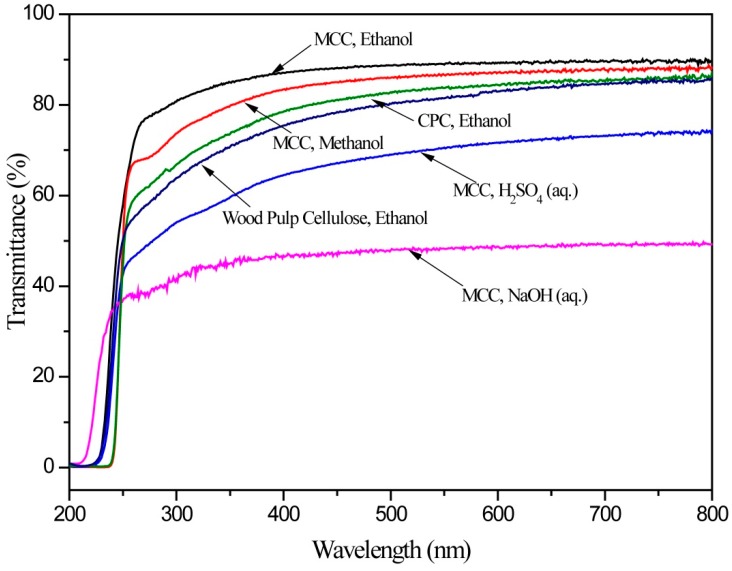
Transmittances of cellulose films.

Light transmittances of films define how transparent they are in proper applications. Figure 8 shows transmittances of the cellulose films over the range of 200–800 nm. Transmittance is above 85% at 800 nm for all films regenerated from ethanol baths, slightly lower than reported for MCC films that had transmittances of over 90% at the same wave number [41,42]. On the contrary, the film regenerated from 5 wt % NaOH (aq.) has the lowest transmittance of less than 50%. Transparency mainly depends on surface morphology, thickness, porosity, and crystal structures of a substance [43,44]. Evidently rough appearances can be observed from the SEM and AFM images for MCC films prepared from 5 wt% NaOH (aq.), and the highest, Ra=7.73, was obtained from the corresponding AFM image. Meanwhile, XRD patterns present the crystalline structure of cellulose. Consequently, both roughness and crystalline structures decreased the transparency of the films.

All regenerated cellulose films surprisingly demonstrated a certain UV shielding between 200–250 nm. In order to determine if the UV shielding was caused by the residual −C=N left over from DBU, the regenerated films were characterized using EDS, but no traces of N were detected. The absorption features at 1750 cm^−1^ in the IRs did not show presence of −C=O. Therefore, the main source of such shielding may be from the amorphous structure of the cellulose membrane, but its mechanism requires further research.

## 4. Conclusions

Cellulosic film is one of important applications of cellulose, which has wide applications in food packaging, separation, etc. In this study, the newly developed solvent system of CO_2_/DBU/DMSO was demonstrated to be a sustainable and efficient solvent system for cellulosic film preparation. The findings demonstrated that the mechanical properties, crystalline structures, transmittances, and topography structures of the as-prepared cellulosic films were significantly correlated to the regeneration solvents. It was found that amorphous and cellulose II films were obtained from alcohol-based and water-based regeneration solvent respectively. The films had good topography structure and excellent transmittances and mechanical properties in the cases of the ethanol and methanol regeneration baths. The mechanical properties of the films significantly improved as the molecular weight of the cellulose increased, which is evidenced by the biggest strength and elongation break of the wood pulp cellulose film being achieved. In other words, this study provided new insights for the preparation of cellulosic films, which has potential applications in packaging and separation fields in the future.

## 5. Patents

The work was patented by Chinese Patent Application: 201910090265.8.

## Figures and Tables

**Table 1:** Regeneration condition and physical properties of the cellulose films ^a^.

System	Time (min)	Cellulose	Strength/(MPa)	Elongation Break/(%)	CrI/(%)	Transmittance/(%)	Young’s Modulus(MPa)
Ethanol	1	MCC	55	6.5	nd.^b^	90	1568
Methanol	1	MCC	42	5.2	nd.	87	3553
NaOH (aq)	5	MCC	27	5	0.16	49	804
H_2_SO_4_(aq)	6	MCC	23	3.1	0.12	74	1897
Ethanol	3	CPC	76	8.2	nd.	85	4352
Ethanol	3	wood pulp	91	9	nd.	85	1770

^a^ Experimental conditions: Cellulose concentration: 5 wt%. ^b^ nd.Stands for no degree of crystallinity.

## References

[1] Llevot A., Dannecker P.K., von Czapiewski M., Over L.C., Soyler Z., Meier M.A. (2016). Renewability is not enough: Recent advances in the sustainable synthesis of biomass-derived monomers and polymers. Chemistry.

[2] Klemm D., Heublein B., Fink H.P., Bohn A. (2005). Cellulose: Fascinating biopolymer and sustainable raw material. Angew. Chem..

[3] Rose M., Palkovits R. (2011). Cellulose-based sustainable polymers: State of the art and future trends. Macromol. Rapid Commun..

[4] Dumitriu C., Voicu S.I., Muhulet A., Nechifor G., Popescu S., Ungureanu C., Carja A., Miculescu F., Trusca R., Pirvu C. (2018). Production and characterization of cellulose acetate—Titanium dioxide nanotubes membrane fraxiparinized through polydopamine for clinical applications. Carbohydr. Polym..

[5] Du X., Zhang Z., Liu W., Deng Y. (2017). Nanocellulose-based conductive materials and their emerging applications in energy devices—A review. Nano Energy.

[6] Han M., Jin X., Yang H., Liu X., Liu Y., Ji S. (2017). Controlled synthesis, immobilization and chiral recognition of carboxylic acid functionalized cellulose tris(3,5-dimethylphenylcarbamate). Carbohydr. Polym..

[7] Cuevas A., Campos B.B., Romero R., Algarra M., Vazquez M.I., Benavente J. (2019). Eco-friendly modification of a regenerated cellulose based film by silicon, carbon and N-doped carbon quantum dots. Carbohydr. Polym..

[8] Dai L., Long Z., Lv Y., Zhang D., Deng H.B., Liu Q. (2015). TEMPO-mediated oxidation of cellulose in carbonate buffer solution. Fibers Polym..

[9] Etemadi H., Yegani R., Babaeipour V. (2016). Study on the reinforcing effect of nanodiamond particles on the mechanical, thermal and antibacterial properties of cellulose acetate membranes. Diam. Relat. Mater..

[10] McCormick C.L., Dawsey T.R. (1990). Preparation of cellulose derivatives via ring-opening reactions with cyclic reagents in lithium chloride/N,N-dimethylacetamide. Macromolecules.

[11] Cai J., Zhang L. (2005). Rapid dissolution of cellulose in LiOH/urea and NaOH/urea aqueous solutions. Macromol. Biosci..

[12] Richard P., Swatloski S.K.S., John D., Holbrey R., Rogers D. (2002). Dissolution of cellose with ionic liquids. J. Am. Chem. Soc..

[13] Qi H., Chang C., Zhang L. (2009). Properties and applications of biodegradable transparent and photoluminescent cellulose films prepared via a green process. Green Chem..

[14] Zhang X., Xiao N., Wang H., Liu C., Pan X. (2018). Preparation and characterization of regenerated cellulose film from a solution in lithium bromide molten salt hydrate. Polymers.

[15] Pang J., Wu M., Zhang Q., Tan X., Xu F., Zhang X., Sun R. (2015). Comparison of physical properties of regenerated cellulose films fabricated with different cellulose feedstocks in ionic liquid. Carbohydr. Polym..

[16] Zheng X., Huang F., Chen L., Huang L., Cao S., Ma X. (2019). Preparation of transparent film via cellulose regeneration: Correlations between ionic liquid and film properties. Carbohydr. Polym..

[17] Wang S., Peng X., Zhong L., Jing S., Cao X., Lu F., Sun R. (2015). Choline chloride/urea as an effective plasticizer for production of cellulose films. Carbohydr. Polym..

[18] Rahman F.A., Aziz M.M.A., Saidur R., Abu Bakar W.A.W., Hainin M.R., Putrajaya R., Hassan N.A. (2017). Pollution to solution: Capture and sequestration of carbon dioxide (CO_2_) and its utilization as a renewable energy source for a sustainable future. Renew. Sustain. Energy Rev..

[19] Wei J., Ge Q., Yao R., Wen Z., Fang C., Guo L., Xu H., Sun J. (2017). Directly converting CO_2_ into a gasoline fuel. Nat. Commun..

[20] Xie H., Yu X., Yang Y., Zhao Z.K. (2014). Capturing CO_2_ for cellulose dissolution. Green Chem..

[21] Zhang Q., Oztekin N.S., Barrault J., De Oliveira V.K., Jérôme F. (2013). Activation of microcrystalline cellulose in a CO_2_-based switchable system. ChemSusChem.

[22] Yang Y., Xie H., Liu E. (2014). Acylation of cellulose in reversible ionic liquids. Green Chem..

[23] Yang Y., Song L., Peng C., Liu E., Xie H. (2015). Activating cellulose via its reversible reaction with CO_2_ in the presence of 1,8-diazabicyclo[5.4.0]undec-7-ene for the efficient synthesis of cellulose acetate. Green Chem..

[24] Chen H., Yang F., Du J., Xie H., Zhang L., Guo Y., Xu Q., Zheng Q., Li N., Liu Y. (2018). Efficient transesterification reaction of cellulose with vinyl esters in DBU/DMSO/CO_2_ solvent system at low temperature. Cellulose.

[25] Song L., Yang Y., Xie H., Liu E. (2015). Cellulose dissolution and in situ grafting in a reversible system using an organocatalyst and carbon dioxide. ChemSusChem.

[26] Jin L., Yu X., Peng C., Guo Y., Zhang L., Xu Q., Zhao Z.K., Liu Y., Xie H. (2018). Fast dissolution pretreatment of the corn stover in gamma-valerolactone promoted by ionic liquids: Selective delignification and enhanced enzymatic saccharification. Bioresour. Technol..

[27] Meenatchi B., Renuga V., Manikandan A. (2017). Cellulose dissolution and regeneration using various imidazolium based protic ionic liquids. J. Mol. Liq..

[28] Nam S., French A.D., Condon B.D., Concha M. (2016). Segal crystallinity index revisited by the simulation of x-Ray diffraction patterns of cotton cellulose Ibeta and cellulose II. Carbohydr. Polym..

[29] Akira I.M.U. (1989). Solid-state CP_MAS carbon-13 NMR study of cellulose polymorphs. Macromolecules.

[30] Tadahisa I.L.I., Jun-Ichi A. (1998). Affinity of hemicellulose for cellulose produced by acetobacterxylinum. Cellulose.

[31] Oh S.Y., Yoo D.I., Shin Y., Kim H.C., Kim H.Y., Chung Y.S., Park W.H., Youk J.H. (2005). Crystalline structure analysis of cellulose treated with sodium hydroxide and carbon dioxide by means of x-Ray diffraction and FTIR spectroscopy. Carbohydr. Res..

[32] Zhang B.X., Azuma J.I., Uyama H. (2015). Preparation and characterization of a transparent amorphous cellulose film. RSC Adv..

[33] Sundberg J., Toriz G., Gatenholm P. (2013). Moisture induced plasticity of amorphous cellulose films from ionic liquid. Polymer.

[34] Jiang G., Huang W., Wang B., Zhang Y., Wang H. (2012). The changes of crystalline structure of cellulose during dissolution in 1-butyl-3-methylimidazolium chloride. Cellulose.

[35] Medronho B., Lindman B. (2015). Brief overview on cellulose dissolution/regeneration interactions and mechanisms. Adv. Coll.Interf. Sci..

[36] Potthast A., Rosenau T., Kosma P., Saariaho A.M., Vuorinen T. (2005). On the nature of carbonyl groups in cellulosic pulps. Cellulose.

[37] Jia N., Li S.M., Ma M.G., Sun R.C., Zhu L. (2011). Green microwave-assisted synthesis of cellulose/calcium silicate nanocomposites in ionic liquids and recycled ionic liquids. Carbohydr. Res..

[38] Zhong L.X., Peng X.W., Yang D., Cao X.F., Sun R.C. (2013). Long-chain anhydride modification: A new strategy for preparing xylan films. J. Agric. Food Chem..

[39] Onwukamike K.N., Tassaing T., Grelier S., Grau E., Cramail H., Meier M.A. (2018). Detailed understanding of the DBU/CO_2_ switchable solvent system for cellulose solubilization and derivatization. ACS Sustain. Chem. Eng..

[40] Liu S., Zhang L., Sun Y., Lin Y., Zhang X., Nishiyama Y. (2009). Supramolecular structure and properties of high strength regenerated cellulose films. Macromol. Biosci..

[41] Liu F., McMaster M., Mekala S., Singer K., Gross R.A. (2018). Grown ultrathin bacterial cellulose mats for optical applications. Biomacromolecules.

[42] Cao Y., Li H., Zhang Y., Zhang J., He J. (2010). Structure and properties of novel regenerated cellulose films prepared from cornhusk cellulose in room temperature ionic liquids. J. Appl. Polym. Sci..

[43] Elsabee M.Z., Abdou E.S. (2013). Chitosan based edible films and coatings: A review. Mater. Sci. Eng. C.

[44] Wang S., Lu A., Zhang L. (2016). Recent advances in regenerated cellulose materials. Prog. Polym. Sci..

